# The C-Terminus of Panusin, a Lobster β-Defensin, Is Crucial for Optimal Antimicrobial Activity and Serum Stability

**DOI:** 10.3390/pharmaceutics15061777

**Published:** 2023-06-20

**Authors:** Roberto Bello-Madruga, Javier Valle, M. Ángeles Jiménez, Marc Torrent, Vivian Montero-Alejo, David Andreu

**Affiliations:** 1 Barcelona Biomedical Research Park, Department of Medicine and Life Sciences, Universitat Pompeu Fabra, 08003 Barcelona, Spain; roberto.bello@uab.cat (R.B.-M.); javier.valle@upf.edu (J.V.); 2 Systems Biology of Infection Lab, Department of Biochemistry and Molecular Biology, Universitat Autònoma de Barcelona, 08193 Cerdanyola del Vallès, Spain; marc.torrent@uab.cat; 3 Department of Biochemistry and Molecular Biology, Center for Pharmaceutical Research and Development, (CIDEM), La Habana 10400, Cuba; 4 Instituto de Química Física Blas Cabrera—CSIC, Serrano 119, 28006 Madrid, Spain; mangeles.jimenez@csic.es

**Keywords:** panusin, β-defensins, antimicrobial peptides

## Abstract

β-defensins are one of the most abundant and studied families of antimicrobial peptides (AMPs). Because of their selective toxicity to bacterial membranes and a broad spectrum of microbicidal action, β-defensins are regarded as potential therapeutic agents. This work focuses on a β-defensin-like AMP from the spiny lobster *Panulirus argus* (hereafter referred to as panusin or PaD). This AMP is structurally related to mammalian defensins via the presence of an αβ domain stabilized by disulfide bonds. Previous studies of PaD suggest that its C-terminus (Ct_PaD) contains the main structural determinants of antibacterial activity. To confirm this hypothesis, we made synthetic versions of PaD and Ct_PaD to determine the influence of the C-terminus on antimicrobial activity, cytotoxicity, proteolytic stability, and 3D structure. After successful solid-phase synthesis and folding, antibacterial assays of both peptides showed truncated Ct_PaD to be more active than native PaD, confirming the role of the C-terminus in activity and suggesting that cationic residues in that region enhance binding to negatively charged membranes. On the other hand, neither PaD nor Ct_PaD were hemolytic or cytotoxic in human cells. Proteolysis in human serum was also studied, showing high (>24 h) t_1/2_ values for PaD and lower but still considerable for Ct_PaD, indicating that the missing native disulfide bond in Ct_PaD alters protease resistance, albeit not decisively. NMR-2D experiments in water agree with the results obtained by circular dichroism (CD), where in SDS micelles, CD showed both peptides adopting an increasingly ordered structure in a hydrophobic environment, in tune with their ability to perturb bacterial membrane systems. In conclusion, while the β-defensin features of PaD are confirmed as advantageous in terms of antimicrobial activity, toxicity, and protease stability, the results of the present work suggest that these same features are preserved, even enhanced, in the structurally simpler Ct_PaD, which must therefore be viewed as a valuable lead for the development of novel anti-infectives.

## 1. Introduction

The ever-growing resistance of microorganisms to antibiotics is compromising the efficacy of anti-infective therapies and has been identified by the World Health Organization (WHO) as one of the main current threats to human health. In this context, there is a pressing need to develop new anti-infective drugs with novel mechanisms of action that preclude or minimize the emergence of resistance [1].

Antimicrobial peptides (AMPs) are increasingly viewed as potential alternatives to conventional antibiotics [2]. These peptides are classified as key constituents of the family of host-defense peptides (HDPs) of both vertebrate and invertebrate organisms [3]. β-defensins, one of the most abundant and widely studied AMPs, were first identified in humans and have later been found in a broad variety of animal types [4].

Panusin, isolated from hemocytes of the spiny lobster *Panulirus argus* (PaD), is representative of those invertebrate peptides, which are related to mammalian β-defensins [5]. PaD is comprised of 39 amino acid residues that fold into a well-described cysteine-stabilized αβ (CSαβ) motif, consisting of an α-helix and three antiparallel β-sheets. These β-sheets are strongly stabilized by three disulfide bonds. PaD has a broad spectrum of microbicidal action against Gram-positive and Gram-negative bacteria and yeast, with no hemolytic effect on human erythrocytes [5]. These properties mark it as an attractive template for the development of anti-infective agents.

Previous work on PaD, using the Membrane Protein Explorer tool (MPEx, https://blanco.biomol.uci.edu/mpex/: accessed on 27 June 2022), has suggested that the C-terminus (Ct_PaD), surface-exposed and scoring the highest hydrophobic moment, might be a determinant of antibacterial activity [5]. Other studies on β-defensin-like peptides also pointed out that the C-terminal region could be a determinant for antimicrobial activity as well as for serum stability and cytotoxicity, with these last two issues being recognized as important challenges to a potential clinical application of AMPs [6].

In light of the above, we decided to assess the influence of the C-terminus of PaD on antimicrobial activity, cytotoxicity, proteolytic stability, and solution 3D structure. To this end, we developed an efficient solid-phase synthesis of PaD, and its truncated analog Ct_PaD, followed by anaerobic oxidation into the native disulfide arrangement. To ensure that the folding had been successful, the oxidized synthetic PaD was tested in HPLC, alongside an authentic natural sample, with satisfactory results. Next, the antimicrobial activities of synthetic PaD and Ct_PaD were assayed, as well as their toxicity toward erythrocytes and human cells. We also determined, for the first time, the stability of both peptides in human serum. Finally, NMR and circular dichroism gave the solution structures of PaD and Ct_PaD. They served as a definitive experimental corroboration of earlier predictions on the key role of the C-terminal segment on antimicrobial activity and serum stability.

## 2. Materials and Methods

### 2.1. Materials

Fmoc amino acids and 2-(1H-benzotriazole-1-yl)-1,1,3,3-tetramethyluronium hexafluorophos-phate (HBTU) were from Iris Biotech GmbH (Marktredwitz, Germany). H-Rink Amide-ChemMatrix resin (0.50 mmol/g substitution) was from PCAS BioMatrix (Quebec, Canada). Solvents for peptide synthesis and HPLC and other reagents were from Thermo Fisher Scientific (Sant Cugat del Vallès, Spain). *Escherichia coli* was obtained from the Coli Genetic Stock Center (BW25113) and *Pseudomona aeruginosa* ATCC 15692, *Acinetobacter baumannii* ATCC 15308, *Staphylococcus aureus* ATCC 12600, *Enterococcus faecium* ATCC 19434, and *Micrococcus luteus* ATCC 15,307 were obtained from the Spanish Type Culture Collection (CECT, Valencia, Spain). Purified horse red blood cells were from Thermo Fisher Scientific. MRC-5 fibroblasts were obtained from the Cytometry and Cell Culture Facility at the Autonomous University of Barcelona and originally purchased from ATCC. Human serum was from Sigma (Madrid, Spain).

### 2.2. Peptide Synthesis

The linear precursors of PaD and Ct_PaD were synthesized as C-terminal carboxamides at 0.1-mmol scale on H-Rink amide ChemMatrix^®^ resin of 0.5 mmol/g substitution (PCAS BioMatrix, Quebec, QC, Canada) in a Prelude instrument (Gyros Protein Technologies, Tucson, AZ, USA) running Fmoc chemistry protocols. Side chains of trifunctional residues were protected with Boc (Lys, Trp), NG-2,2,4,6,7-pentamethyldihydrobenzofuran-5-sulfonyl (Arg), tert-butyl (Ser, Asp, Glu), and trityl (Asn, Cys) groups. Couplings used 8-fold molar excess of both Fmoc-amino acid and HBTU, in the presence of 16-fold molar excess of DIEA in N,N-dimethylformamide (DMF). Fmoc was removed with piperidine/DMF (20:80 *v*/*v*) followed by DMF washes. After chain assembly, full deprotection and cleavage from the resin were performed with TFA/H_2_O/TIS/DODT (94:2,5:2,5:1 *v*/*v*, 90 min) at room temperature. Crude peptides were isolated from the TFA solution by precipitation with chilled diethyl ether and centrifugation at 5000 rpm for 2 × 10 min at 4 °C, and then dissolved in water and lyophilized.

### 2.3. RP-HPLC and LC-MS

Analytical RP-HPLC was performed on an LC-2010 instrument (Shimadzu, Kyoto, Japan) using a Luna C18 column (4.6 × 50 mm, 3 µm; Phenomenex, Torrance, CA, USA), eluted with linear gradients of solvent B (0.036% TFA in MeCN) into solvent A (0.045% TFA in H_2_O) over 15 min at a 1 mL/min flow rate, with UV detection at 220 nm.

Preparative peptide purification was performed by RP-HPLC on Shimadzu LC-20AP equipment, using an Aeris Peptide XB-C18 column (250 × 21.2 mm, 5 µm; Phenomenex), eluted with a linear gradient of solvent B (0.1% TFA in MeCN) into A (0.1% TFA in H_2_O) over 30 min at a 20 mL/min flow rate, with UV detection at 220 nm.

LC-MS was performed on a 2010EV instrument (Shimadzu) fitted with an Aeris Widepore XB-C18 column (150 × 4.6 mm, 3.6 µm, Phenomenex), eluting with linear gradients of solvent B [0.08% formic acid (FA) in ACN] into A (0.1% FA in H_2_O) over 15 min at a 1 mL/min flow rate. Fractions of >95% HPLC homogeneity and the expected mass (4260.5 Da and 2801.6 Da for PaD and Ct_PaD precursors, respectively) were pooled and lyophilized.

### 2.4. Anaerobic Oxidative Folding

The hexa and tetrathiol precursors (PaD and Ct_PaD, 10 mg of each) were dissolved in 0.1 M NH_4_OAc and 1 M Gd-HCl buffer (pH 8.2, deaerated by N_2_ bubbles) at a 10^−5^ M final concentration in the presence of reduced (GSH) and oxidized glutathione (GSSG) (1:100:10 peptide/GSH/GSSG, molar ratio). The reaction was allowed to run at room temperature, under a N_2_ stream with gentle agitation, and monitored every 2 h by HPLC. At each time point, 200 µL of the mixture was neutralized with 20 µL of acetic acid (HOAc, 10% *v*/*v*) and analyzed by RP-HPLC and LC-MS (conditions as described above). After 24 h, when the reaction was completed, the mixture was acidified to pH 5 with HOAc and applied by a peristaltic pump for desalting onto a 20 mL Strata C18-E cartridge (Phenomenex) preconditioned with wetting buffer (0.1% TFA in MeCN, 4 × 20 mL) and equilibration buffer (0.1% TFA in H_2_O, 4 × 20 mL). The cartridge was washed (4 × 20 mL) with the equilibration buffer to remove the salts, and the fully oxidized peptide was then eluted with 0.1% TFA in 50% MeCN/H_2_O and lyophilized. Next, synthetic PaD or Ct_PaD (see Table 1) were further purified by semi-preparative RP-HPLC on an Aeris XB-C18 column (250 × 10.0 mm, 5 µm; Phenomenex), using a 10–50% linear gradient of MeCN into 0.1% TFA in H_2_O over 30 min at a 7 mL/min flow rate. Fractions with >95% HPLC homogeneity and the expected mass were collected, lyophilized, and used in subsequent experiments.

### 2.5. Minimum Inhibitory Concentration (MIC) and Minimum Bactericidal Concentration (MBC)

Antimicrobial activities of both PaD and Ct_PaD were determined in a modified microdilution assay as described previously for other β-defensins such as peptides [7,8]. The assay was performed in sterile polypropylene 96-well plates that prevent peptide binding (Greiner, Frickenhausen, Germany). From a bacterial isolate, a single colony was seeded in Mueller Hinton Broth (MHB, Condalab, Torrejón de Ardoz, Spain) and allowed to grow at 37 °C in an incubator with orbital shaking at 250 rpm overnight. Subsequently, a new bacterial culture was seeded from the overnight culture and allowed to grow to an OD of 0.5 at 600 nm. The bacterial suspension was diluted to a final concentration of 5 × 10^5^ CFU (colony formation unit)/mL in sterile 10 mM phosphate buffer (PB: 2.46 mM NaH_2_PO_4_, 7.5 mM Na_2_HPO_4_, pH 7.4), used as the working culture. Next, 50 μL of 2-fold serial dilutions of each peptide in PB were placed on the plates to provide a final 0.097–100 µM concentration range after which 50 μL of working culture was applied to each well. The plate was first incubated for 2 h at 37 °C, then 100 μL of MHB 2× was added to each well and was incubated at 37 °C overnight with shaking. The growth of bacteria was measured by the optical density at 600 nm in a microplate reader (Tecan, Männedorf, Switzerland). The minimum inhibitory concentration (MIC) was defined as the minimum peptide concentration in which the microorganism is unable to grow. The results are the average of three independent studies. To determine the MBC, 50 μL from all wells were seeded into Petri plates and incubated at 37 °C for 24 h. MBC was defined for each peptide as the lowest concentration with no bacterial growth detected.

### 2.6. Hemolytic Activity

Both peptides were tested for hemolytic activity on horse red blood cells (RBCs), as described earlier [9]. Briefly, fresh RBCs in PBS (pH 7.4) were first centrifuged 3 × 1500× *g* for 5 min, then diluted 1:10 with PBS to yield an RBC concentration of ~5 × 10^8^ cells/mL. RBCs were then incubated for 1 h at 37 °C with 2-fold serial dilutions of PaD or Ct_PaD covering a 0.24–250 μM concentration range. Next, 0.1% TritonX100 and PBS were used as positive and negative controls, respectively. After incubation, samples were centrifuged at 1500× *g* for 10 min at room temperature and the supernatant (60 μL) was transferred to a flat-bottomed 96-well polypropylene microplate (Greiner, Frickenhausen, Germany). Hemoglobin release was then measured as OD at 540 nm in a VICTOR3 microplate reader (Perkin Elmer, Waltham, MA, USA). Percent hemolysis was calculated as indicated below (Equation (1)). Results are the average of three independent replicates.

% Hemolysis = [OD peptide _540_] − [OD PBS _540_]/[OD triton _540_] − [OD PBS _540_] × 100 (1)

### 2.7. Cytotoxicity in Mammalian Cells

MRC-5 cells were cultured in Eagle’s minimum essential medium (MEMα), supplemented with 10% (*v*/*v*) fetal bovine serum (FBS) and a 1% penicillin (100 IU/mL)/streptomycin (100 μg/mL) solution, and maintained at 37 °C in a humidified atmosphere with 5% CO_2_. After cells were grown, they were seeded into 96-well plates at 3 × 10^4^ cells/well and cultured overnight to adhere to the plate. Subsequently, adherent cells were incubated for 4 h under standard conditions (5% CO_2_, 37 °C) with 2-fold serial dilutions of each peptide in a 1.56–200 μM concentration range. Following incubation, the peptide-containing medium was replaced with MEM supplemented with FBS and 3-(4,5-dimethylthiazol-2-yl)-2,5 diphenyltetrazolium bromide (MTT) at 0.4 mg/mL, and the cells were further incubated for 120 min. Formazan crystals in live cells were detected after disruption with 200 μL of DMSO by OD measurement at 570 nm in a Tecan microplate reader. TritonX100 (1%) and PBS were used as positive and negative controls, respectively. Data are the average of three independent replicates.

### 2.8. Serum Stability

Peptide samples (500 μL, 500 μM in H_2_O) were mixed and incubated with 500 μL of human serum (Sigma-Aldrich, St. Louis, MO, USA) for 24 h at 37 °C. Aliquots (120 μL) were taken at 0, 30, 120, 360, 720, and 1440 min and treated with 20 μL of trichloroacetic acid (15% (*v*/*v*) in H_2_O). The suspension was centrifuged for 30 min at 13,000 rpm, 4 °C, and the supernatant was analyzed by RP-HPLC as described above. The unaltered peptide was determined by RP-HPLC peak integration, expressed as the percent of the amount at t0, and data were fitted to a one-phase exponential decay model using GraphPad Prism 8.01 to estimate half-life time (t_1/2_). Results are the average of three independent runs.

### 2.9. NMR Spectroscopy

NMR samples were prepared by dissolving the lyophilized peptides in 0.5 mL of either H_2_O/D_2_O (9:1 *v*/*v*) or pure D_2_O. Peptide concentrations were approximately 1 mM. Sodium 2,2-dimethyl-2-silapentane-5-sulfonate was added as an internal reference for 1H chemical shifts. pH was measured with a glass microelectrode (not corrected for isotope effects) and adjusted, if necessary, by adding minimal amounts of NaOD or DCl.

NMR spectra were acquired on a Bruker AVNEO-600 spectrometer operating at a 600.13 MHz proton frequency and equipped with a cryoprobe. The probe temperature was calibrated using a methanol sample. As previously reported [10], standard pulse sequences were used to record 1D and 2D spectra, i.e., double-quantum filtered phase-sensitive correlated spectroscopy (DQF-COSY), total correlated spectroscopy (TOCSY), nuclear Overhauser enhancement spectroscopy (NOESY), and ^1^H−^13^C heteronuclear single quantum coherence spectra (HSQC), which were processed using the TOPSPIN program (Bruker Biospin, Karlsruhe, Germany). Mixing times for TOCSY and NOESY spectra were 60 and 150 ms, respectively. ^1^H−^13^C HSQC spectra were recorded at natural abundance. The ^13^C δ-values were indirectly referenced using the IUPAC-recommended ^13^C/^1^H ratio 0.25144953 [10].

^1^H and ^13^C chemical shifts for synthetic PaD and Ct_PaD in an aqueous solution were assigned using the NMRFAM-SPARKY software [11] and following a standard sequential analysis [12] of 2D DQF-COSY, TOCSY, and NOESY spectra, which were examined in combination with the corresponding 2D ^1^H−^13^C HSQC spectra. The assigned chemical shifts have been deposited at the BioMagResBank (http://www.bmrb.wisc.edu: accessed on 9 May 2023) under accession codes 51942 (PaD) and 51941(Ct_PaD).

Temperature coefficients (Δδ/ΔT, ppm.K^−1^), which were calculated for the amide HN protons of PaD from the difference between their chemical shifts measured at 5 °C and 25 °C, are listed in Table S5.

Structure calculations were performed using the standard iterative protocol for automatic NOE assignment implemented in the CYANA 3.98 program [13]. The algorithm comprises seven cycles of combined automated NOE assignment and structure calculation, with 100 conformers computed per cycle. The experimental input data include the lists of assigned chemical shifts and integrated NOE cross-peaks present in the 150 ms NOESY spectra, plus the ϕ and ψ dihedral angle restraints. The automatic integration subroutine of the NMRFAM-SPARKY software [11] was used for integrating the NOE cross-peak. The dihedral angle restraints were derived from ^1^H and ^13^C chemical shifts using the TALOSn web server [14,15]. Additional upper and lower limit restraints were included for the disulfide bonds. In the case of PaD, upper and lower limit restraints were added for those HN protons that show temperature coefficients that are small in absolute value (|Δδ/ΔT| < 4.5 × 10^−3^ ppm; see Table S5) and were hydrogen-bonded in the structure calculated without hydrogen-bond restraints. The final NMR structures for each peptide correspond to the ensemble of the 20 conformers with the lowest target function value, which were visualized using the MOLMOL program [16]. Structures are available upon request from the authors.

### 2.10. Circular Dichroism Spectroscopy (CD)

CD experiments were performed at room temperature on a Jasco CD-J-815 spectropolarimeter using quartz cells of 1.0 mm thickness. Peptides were dissolved in H_2_O and in a 1–30 mM SDS concentration range to simulate a membrane environment, with a final 50 μM concentration in all cases. Spectra were recorded in the 190–250 nm range and accumulated 15× to improve the signal-to-noise ratio. Baselines of samples without peptides in either water or micelle suspensions were subtracted from each reading to calculate the peptide contribution. Primary data were processed in OriginPro 2022, with the Savitzky–Golay method used for smoothing. The molar mean residue ellipticity [θ] was calculated as follows (Equation (2)):

[θ] (deg cm^2^ dmol^−1^) = θ(mdeg)/(10 × L × [M] × N) (2)

where L is the path length (cm), [M] is the concentration (mol/L), and N is the number of peptide bonds, i.e., the number of residues minus one.

## 3. Results and Discussion

### 3.1. Peptide Design and Synthesis

**Figure 1:**
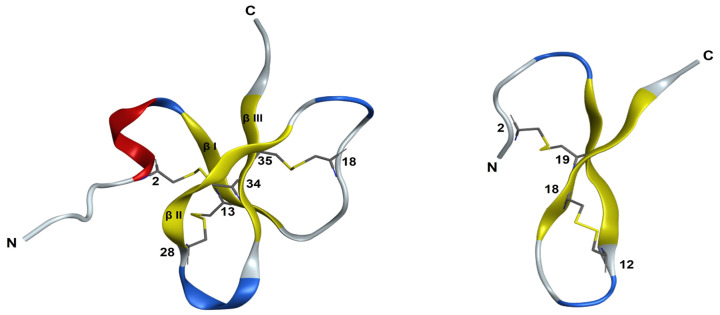
Three-dimensional structure of PaD (**left**) and Ct_PaD (**right**) as predicted by AlphaFold [17]. https://colab.research.google.com/github/deepmind/alphafold/blob/main/notebooks/AlphaFold.ipynb: accessed on 12 April 2022. Tertiary structures are represented using MOE software: Helix is red, β-strand is in yellow, turn is in blue, and ribbon loop is in gray. Disulfide bonds are displayed in yellow.

The 3D structure of PaD (Figure 1) is composed of a three-strand core of antiparallel β-sheets stabilized by three disulfide bonds (Cys1–Cys5; Cys2–Cys4; Cys3–Cys6) and an α-helical domain towards the N-terminus. This pattern was deduced from its alignment with other β-defensins and predictions by homology modeling [5]. Accordingly, the C-terminal region is amidated (Tyr39) and rich in cationic residues unevenly distributed and conferring net charge 4+ to the molecule. On a water/lipid bilayer interface, the segment with the highest hydrophobic moment, residues 15–34 (SSYCPYGNRLNYFCPLGRTC, MH = 2.94), spans the entire βI loop and extends into most of the βII loop (Figure 1). It appears exposed on the molecular surface, which suggests a possible role in membrane interaction hence antimicrobial activity.

To corroborate the hypothesis that the C-terminal of PaD retains the main structural determinants of antibacterial activity, we designed and made a truncated analog (Ct_PaD) with four Cys residues, including those in the native Cys3-Cys6 disulfide of PaD (Table 1).

### 3.2. Synthesis and Oxidative Folding of PaD and Ct_PaD

Linear versions of PaD and Ct_PaD were readily obtained by solid-phase synthesis (SPPS) using Fmoc-tBu methodology as described before [18]. After resin cleavage and preparative RP-HPLC, the fully reduced form of peptides was shown to be highly pure. LC-MS provided expected masses of 4265 and 2805.6 Da for PaD and Ct_PaD, respectively (Table S1 and Figure S1).

Disulfide bridges were generated by the oxidative folding of the linear precursors (hexathiol and tetrathiol, respectively) of PaD and Ct_PaD, under anaerobic thermodynamically controlled conditions [18]. Both oxidation reactions were fast and clean. They led to major end products (monitored for stability over 24 h, Figure S2) with 6 and 4 Da mass differences over the precursor, after 4 and 6 h, respectively.

**Figure 2:**
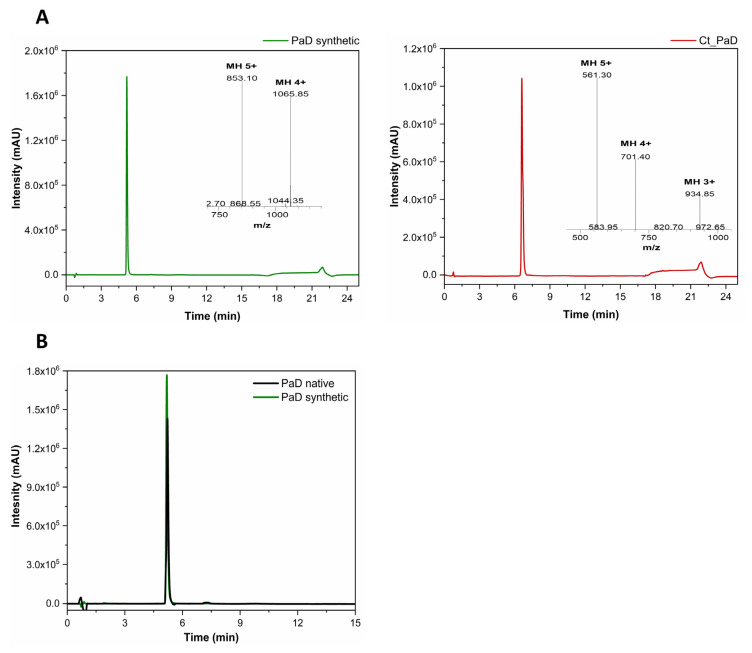
Oxidative folding of PaD and Ct_PaD. (**A**) RP-HPLC elution profile of purified peptides, with (inset) the respective LC-MS spectrum: [M + 5H]^5+^ (*m*/*z* 853.10) and [M + 4H]^4+^ (*m*/*z* 1065.85) for PaD (**Left**), and: [M + 5H]^5+^ (*m*/*z* 561.30), [M + 4H]^4+^ (*m*/*z* 701.40) and [M + 4H]^3+^ (*m*/*z* 934.85) for Ct_PaD (**Right**). (**B**) Overlaid RP-HPLC elution profiles of natural and synthetic PaD samples.

For PaD, the LC-MS data made it plausible to assume that three native disulfide bonds (Cys6-Cys34, Cys13-Cys28, and Cys18-Cys35) had formed. This hypothesis was corroborated by two additional findings: (1) Synthetic PaD co-eluted with natural PaD under the same RP-HPLC conditions (Figure 2B) and (2) the ^1^H,^1^H-TOCSY NMR spectra of natural and synthetic PaD showed essentially the same cross-peaks, (see Supplementary Information, Figure S4). On the strength of comparable findings in similar defensin peptides [18], a native folding was adjudicated to be synthetic PaD.

For Ct_PaD, the LC-MS data again indicated a fully oxidized product, but in the absence of a natural standard, no definite assignment of disulfide connectivity could be made. Until reliable structural evidence was further obtained (see Structural Analysis below), we tentatively assumed, as predicted by AlphaFold (Figure 1), that a native-like Cys18-Cys35 bridge had formed and that it had, in turn, induced a non-native Cys28-Cys34 pairing.

The HPLC retention times of PaD and Ct_PaD reflect some differences in hydrophobic character. Specifically, the lower retention time of PaD (Figure 2A and Table S1) suggests a relatively weaker interaction (adsorption-partition) with the C18 matrix, in tune with the hydropathicity index, among others (Table 2). Such differences in hydrophobicity might influence both antimicrobial and cytotoxic activity, which were therefore assessed next.

### 3.3. Antimicrobial Activity

Synthetic PaD and Ct_PaD were tested for antimicrobial activity, expressed as the minimal inhibitory concentration (MIC), against Gram-negative (*Escherichia coli*, *Pseudomonas* sp., *Acinetobacter baumannii*) and Gram-positive (*Staphylococcus aureus*, *Enterococcus faecium*, and *Micrococcus luteus*) reference strains (Table 3). PaD showed roughly equivalent activity against Gram-negative and Gram-positive strains, as previously observed for the native peptide [5]. In contrast, the shorter Ct_PaD showed higher activity than PaD against all bacteria. Both peptides showed minimal bactericidal concentrations (MBC), within the same range as the MICs (Table 3), evidencing their bactericidal character.

The mechanism of action of AMPs is primarily recognized to involve the initial electrostatic interaction of the peptide with the target membrane. Differences in activity between PaD and Ct_PaD can therefore be related to the increase in net positive charge of Ct_PaD, due to the lack of Asp5, as reflected in the higher Boman index [20,21].

Interestingly, PaD retains its antimicrobial activity at high salt concentrations [5], despite its lower net charge. Thus, hydrophobicity and amphipathicity might play a role in the antimicrobial activity. If so, the higher hydrophobic moment (MH) of Ct_PaD relative to PaD (Table 2) may enhance antibacterial activity. In short, Ct_PaD appears to retain the main structural features that make PaD active against bacterial membranes. This is in tune with earlier studies on human β-defensin 1 (HBD-1) and 3 (HBD-3) C-terminal peptides, similarly showing that, despite retaining only one of the three disulfides, the truncated versions were essentially equipotent to the parental peptides, suggesting that the increased positive charge and amphipathic character improved activity [22,23]. In our case, the structural arrangement resulting from the loss of at least two of the canonic disulfide bridges not only has no detrimental effects but actually boosts antimicrobial activity while preserving proteolytic resistance [6,7,21].

### 3.4. Hemolytic Activity and Cell Viability

Since selective toxicity to bacteria over eukaryotic membranes is a key feature of successful AMPs, antibacterial activity is best evaluated alongside cytotoxicity [24].

**Figure 3:**
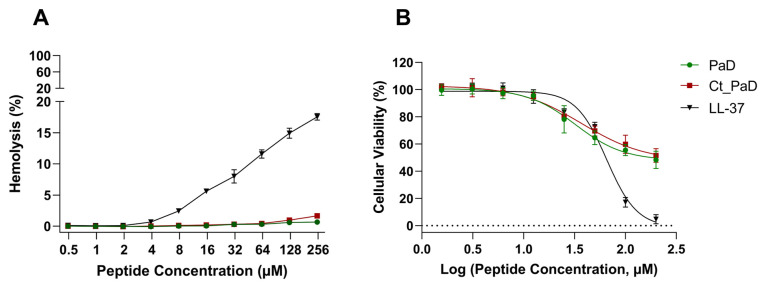
Toxicity analysis. (**A**) Hemolytic activity in horse red blood. (**B**) Viability of hepatic cells (MRC-5) upon peptide treatment for 4 h. Data points for PaD and Ct_PaD peptides are depicted in green and red. Results are the average of three replicates. LL-37 was used as a reference peptide.

The cytotoxicity of PaD and Ct_PaD was first determined as horse red blood cell lysis, monitored by hemoglobin release, at 540 nm [25]. PaD, Ct_PaD, and the reference peptide LL-37 were tested up to 250 μM. The results in Figure 3A and Table 3 confirm the absence of hemolytic activity in both synthetic PaD and Ct_PaD, as previously shown for the native material [5].

Hemolysis data were complemented by an MTT cytotoxicity assay on MRC-5 cells. As shown in Figure 3B, the low toxicity of PaD and Ct_PaD was confirmed, with IC_50_ of 145 and 175 µM (Table 3), respectively, significantly better than the LL-37 control (IC_50_ 65 μM), an established AMP. In particular, the low cytotoxicity of Ct_PaD corroborates that the cationic C-terminus is not harmful to eukaryotic cells [6]. Altogether, the encouraging cytotoxicity data portray both PaD and Ct_PaD as valuable antimicrobial scaffolds, meriting further study as therapeutic leads.

### 3.5. Serum Stability

Any prospective study for peptide-based drugs must soon deal with their proteolytic (un)stability, a significant hurdle in therapeutic application [26,27,28]. In light of this, we evaluated the proteolytic susceptibility of PaD and Ct_PaD in human serum.

**Figure 4:**
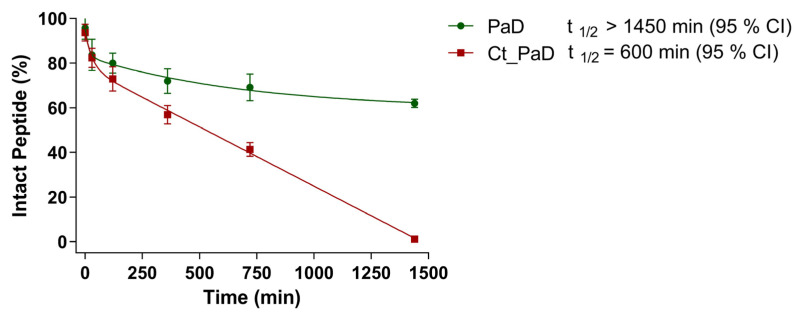
Stability of peptides in human serum. Peptide extinction curves over time of PaD and Ct_PaD were obtained by integration of chromatogram peaks in Figure S3. The t_1/2s_, estimated by experimental data fitting to an exponential decay model, are the mean of three experiments, with a 95% CI.

Kinetic data (Figure 4) from LC-MS analysis of the serum stability of PaD and Ct_PaD (Figure S3) showed remarkable (t_1/2_ > 1450 min) and adequate (t_1/2_ = 600 min) stabilities for PaD and Ct_PaD, respectively. That both peptides are C-terminal amides, thus shielded against carboxypeptidases [29,30], reasonably accounts for the substantial lifetimes. In addition, the presence of an N-terminal α-helix [29] and the highly compact 3D structure of PaD (Figure 1) underpin the substantial additional stability [23,31,32]. The results also reinforce the notion that for a bioactive peptide, a cyclic structure is an obvious asset vis-à-vis protease stability, hence improved pharmacokinetic/pharmacodynamic profiles [33,34,35].

### 3.6. NMR Structural Study

Two-dimensional NMR studies using four types of experiments (DQF-COSY, TOCSY, NOESY, and ^1^H−^13^C HSQC) were performed for PaD and Ct_PaD to gain insight into their 3D structures.

The assignment of ^1^H and ^13^C chemical shifts allowed us to conclude that the rotamer state of the two X-Pro bonds in both PaD and Ct_PaD was trans (small difference in ^13^Cβ and ^13^Cγ chemical shifts [36,37]; Table S2, Supplementary Information), also confirmed by the sequential NOEs between the Pro H_δδ_ protons and the Hα proton of the preceding residue.

That the canonic disulfide pattern was indeed formed was corroborated by (i) the ^13^Cβ chemical shifts of Cys residues, all >35 ppm (vs. <32 ppm for reduced Cys residues, Table S3; [38]); and (ii) coincident cross-peaks in the 2D ^1^H,^1^H-TOCSY spectra of natural and synthetic PaD, despite the low concentration available for the natural version (Figure S4).

To further corroborate the disulfide pairing, a preliminary structure calculation in the CYANA program [13] was performed using NMR-derived NOE and dihedral angle restraints but did not include any disulfide pairing. In the resulting ensemble of 20 conformers, the 6-34 and 18-35 disulfide bonds are present in 14 and 17 of the conformers, respectively, whereas for Cys13-Cys28, the side-chains were relatively close though did not define a disulfide bond (Figure S5). This corroborates the folding pattern anticipated above and also matches the structure predicted by ab-initio methods such as AlphaFold (Figure 1).

Next, the 3D structure of PaD was determined from the experimental NMR restraints (see above), including those required for disulfide pairing. Except for a short, disordered N-terminal region (residues 1–4), the resulting structure (Figure S6) is well-defined (RMSD for backbone atoms of residues 5-38 is 0.5 ± 0.2 Å; see Table S4) and consists of a short α-helical region spanning residues 6–9, and a two-stranded antiparallel β-sheet with topology β1–β3, as schematically shown in Figure S7; (β1: 12–14; β3:33–35). The loop connecting these two β-strands contains several turns (Promotif analysis; ref.), i.e., γ -turn (16–18; SYC), β-turn I (19–22; PYGN), β-turn I (20–23; YGNR), β-turn I (19–22; PYGN), and β-turn II (29–32; PYGN), plus an extended region in which residues 23–24 and 27–28 face β3 (therefore denoted as β2; see Figure S7). The conformational shifts of the H_α_ protons and C_β_ carbons agree with these elements of secondary structure (see Figure S8). Since most hydrogen-bonded amide HN groups showed temperature coefficients small in absolute value (see Table S5), and amide HN protons of S12 and C35, hydrogen-bonded in the β-sheet, were slow-exchanging (observed for >20 h after dissolving lyophilized PaD in D_2_O), it was decided to refine the PaD structure by incorporating hydrogen-bond restraints for these HN protons.

**Figure 5:**
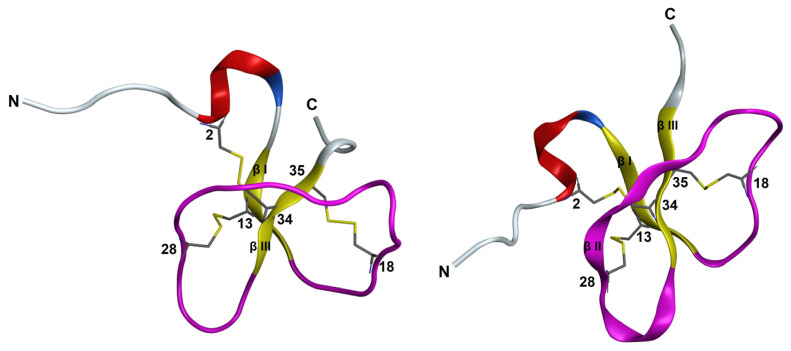
Ribbon representation of the PaD structure. (**Left**) Lowest target function conformer of the final NMR structure (**Right**) Alpha-fold predicted structure. Disulfide bonds are displayed in yellow. Loops 15–32 are highlighted in magenta.

While the resulting structure was essentially identical to that calculated without including hydrogen bonds (Figure S6), it had more phi and psi angles in the most favored region of the Ramachandran diagram (Table S4) and the RMSD for backbone atoms of residues 5–38 was 0.5 ± 0.2 Å smaller; Table S4). The comparison of the final structural ensemble and the AlphaFold predicted model is quite satisfactory overall (Figure 5), with the main difference being that an β2-strand in the AlphaFold-predicted model does not appear in the NMR-derived structure.

In contrast to PaD, non-sequential NOE cross-peaks could not be observed in the ^1^H,^1^H-NOESY spectrum of Ct_PaD, which suggests a primarily disordered structure. The conformational shifts of the H_α_ protons and C_β_ carbons (see Figure S8), which lie mostly in the range expected for random coil peptides, also indicate that Ct_PaD is not as well-folded as PaD. The CYANA program [13] was used to calculate the structure, using distance restrictions derived from intraresidual and sequential NOE cross-peaks and dihedral angle restrictions derived from ^1^H and ^13^C chemical shifts. The calculation yielded a structure (Figure S9) with a poorly defined N-terminal region (approximately residues 1–11) and a more ordered C-terminal region (approximately residues 12–22). In this resulting structural ensemble, the disulfide bonds could not be placed, except for a single conformer with a 12–18 disulfide pairing.

**Figure 6:**
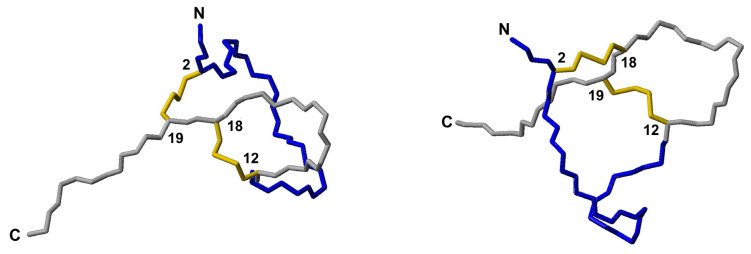
Ct_PaD structure. Lowest target function conformers of the NMR structures were calculated for disulfide pairing 2–19 and 12–18 (**left**) and for disulfide pairing 2–18 and 12–19 (**right**). Backbone atoms are shown in blue and grey neon for the disordered and ordered regions, respectively. Disulfide bonds are displayed in yellow.

Attempts were made to explore the compatibility of the three possible disulfide pairings, i.e., 2–12 and 18–19, 2–18 and 12–19, and 2–19 and 12–18, with experimental distances and angle restraints. Those for the 2–12 and 18–19 pairing caused large violations of distance and angle restraints, so it was discarded. The other possible arrangements were both compatible with experimental restraints (Figure 6). Both ensembles exhibited a poorly defined N-terminal (residues 1–11) and a more ordered C-terminal region (Figure S10), with residues 13–16 and 17–22 displaying turn-like and extended structures, respectively. These ordered conformations were reminiscent of the turn and C-terminal β-strand in the AlphaFold-predicted structure (Figure 1). Structure quality was slightly better for the 2–19 and 12–18 pairing (Table S4), which matches the AlphaFold prediction, but the difference between 2–18 and 12–19 was too small to be conclusive.

### 3.7. Analysis of Secondary Structure by Circular Dichroism

**Figure 7:**
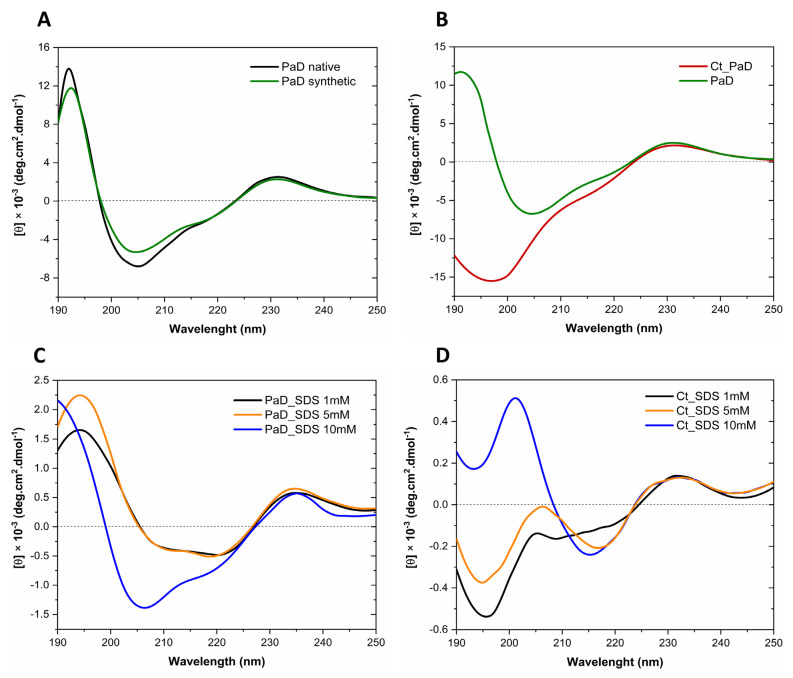
(**A**) Overlaid CD spectra of native and synthetic PaD. (**B**) CD spectra of PaD and Ct_PaD in water. CD spectra of PaD (**C**) and Ct_PaD (**D**) in membrane micro-environment with SDS monomers and micelles.

The overlaid CD spectra of synthetic and natural PaD in H_2_O are shown in Figure 7A. The ellipticity (θ) maxima between 190 and 195 nm suggest a very similar helical content [28]. Secondly, the negative bands at 206 and 218 nm for both peptides suggest a combination of helical and β-sheet conformation [28]. Finally, a positive maximum of lower θ around 230 nm indicates aromatic residues and disulfide bonds (32) for both peptides. In sum, CD results for both peptides support the correct folding by the synthetic peptide [12] and reinforce the NMR data. For its part, the CD spectrum of Ct_PaD in H_2_O (Figure 7B) displays a predominant random structure, with a minimum below 200 nm [28] and a maximum of approximately 230 nm due to the disulfide bonds. From these data, it seems clear that adopting an organized structure in an aqueous solution requires the complete PaD sequence, as found for other β-defensins [29,30].

To analyze PaD and Ct_PaD structures in a membrane-like environment, we used SDS at concentrations above and below the critical micellar concentration (CMC, 7–10 mM) [39]. As seen in Figure 7C, PaD exhibits a helical structure in the presence of SDS monomers (1 and 5 mM), with a maximum between 190 and 195 nm and a minimum of 206 nm and 222 nm. In micelles (10 mM SDS), the relative intensities of the 206 nm and 222 nm minima are inverted, with the latter slightly shifted toward 218 nm, in tune with other described α + β fold CD signatures [5,40].

Ct_PaD shows no significant changes in SDS monomers (Figure 7D) relative to the random structure in H_2_O. A maximum above 200 nm and a minimum above 215 nm appearing when approaching the CMC (5 mM) are more evident in the presence of micelles (10 mM SDS), suggesting a transition to β-sheet (stronger θ minimum above 215 nm and switch from minimum to maximum between 195 and 200 nm). While not exactly confirming the expected folding pattern, this would agree with the structure predicted by AlphaFold (Figure 1), implying that the CD signature is compatible with the hypothesized Ct_PaD disulfide pattern.

## 4. Conclusions

In this work, we have shown that the main structural determinants of panusin (PaD) are preserved in its terminal carboxyl (Ct_PaD). Both peptides were successfully obtained by chemical synthesis. Antibacterial assays of both natural and synthetic PaD showed identical activity for both peptides, providing preliminary evidence of structural identity. Ct_PaD showed 2–3-fold more activity than its parent PaD against Gram-positive and Gram-negative bacteria. Interestingly, this increased activity does not affect toxicity; hence, the potential therapeutic window is improved. While PaD is extremely stable to human serum proteases, Ct_PaD is more vulnerable though still considerably resistant, suggesting that the missing disulfide is important but not decisive. Structural analysis of native PaD, both natural and synthetic, by NMR and CD confirmed that the synthetic peptide is correctly folded in a canonical motif αβ that preserves the arrangement of disulfide bonds Cys1–Cys5, Cys2–Cys4, and Cys3–Cys6, typical of the β-defensin peptides. On the other hand, while NMR analysis confirms Cys connection and CD studies indicate structural rearrangement of Ct_PaD in a membrane environment, we cannot ensure that the predicted disulfide arrangement prevails. Our findings confirm Ct_PaD as the minimal structural motif in PaD with relevant antimicrobial properties, hence a prospective anti-infective peptide lead.

## Figures and Tables

**Table 1:** Primary structures and analytical data for synthetic PaD and Ct_PaD.

ID	Sequence	Cys Pairing	Theoretical Mass (Da) ^1^	Experimental Mass (Da) ^1^	Purity (HPLC, %)
PaD	SYVGDC_1_GSNGGSC_2_VSSYC_3_PYGNRLNYFC_4_PLGRTC_5_C_6_RRSY-amide	C_1_–C_5_ C_2_–C_4_ C_3_–C_6_	4259.76	4260.5	98.7
Ct_PaD	YC_3_PYGNRLNYFC_4_PLGRTC_5_C_6_RRSY-amide	C_3_–C_6_ C_4_–C_5_	2801.25	2801.6	99.8

^1^ Oxidized form.

**Table 2:** Physical-chemical properties of PaD and Ct_PaD.

ID	Net Charge ^a^	pI ^b^	Aliphatic Index ^c^	Hydrophobic Moment ^d^	Boman Index (kcal/mol) ^e^
**PaD**	4+	8.64	34.87	1.86	1.99
**Ct_PaD**	5+	9.20	33.91	2.56	2.59

^a^ Net charge (C-terminus amidation included); ^b^ Isoelectric point; ^c^ Aliphatic Index based on ExPASy’s ProtParam server [19] ^d^ Hydrophobic moment in water/lipid bilayer interface determined by MPEx; ^e^ Boman index represents the binding potential of peptides on bacterial membranes [20].

**Table 3:** Antimicrobial, hemolytic, and cytotoxic activities of PaD and Ct_PaD.

		Peptide		
		PaD	Ct_PaD	LL-37
**MIC/MBC (µM)**	*Escherichia coli*	12.5/12.5	3.1/3.1	1.6/3.1
	*Pseudomonas aeruginosa*	25/25	6.3/6.3	1.6/1.6
	*Acinetobacter baumannii*	25/25	6.3/12.5	6.3/6.3
	*Staphylococcus aureus*	25/25	3.1/6.3	25/25
	*Enterococcus faecium*	25/25	6.3/6.3	1.6/3.1
	*Micrococcus luteus*	12.5/12.5	3.1/3.1	1.6/3.1
**Toxicity**	Hemolysis % (250 µM peptide)	0.6 ± 0.02	1.7 ± 0.08	17.5 ± 0.3
	MRC-5 cytotoxicity (IC_50_, µM)	145 ± 1.7	175 ± 1.5	65 ± 0.7

## Data Availability

Not applicable.

## Supplementary Materials

The following supporting information can be downloaded at: https://www.mdpi.com/article/10.3390/pharmaceutics15061777/s1, Figure S1: RP-HPLC elution profile of purified peptides; Figure S2: Oxidative folding of peptides; Figure S3: RP-HPLC chromatograms of peptides in human serum; Figure S4: TOCSY spectra of natural and synthetic PaD; Figure S5: PaD structure calculated from NMR experimental without restrains for disulfide bond pairings; Figure S6: PaD structures calculated from NMR experimental restraints; Figure S7: Schematic representation of the antiparallel β-sheet present in the NMR structure of synthetic PaD; Figure S8: Conformational shifts of PaD and Ct_PaD as a function of sequence; Figure S9: Ct_PaD structures calculated from NMR experimental without restrains for disulfide bond pairings; Figure S10: Ct_PaD structures calculated from NMR experimental restraints; Table S1: Peptide analytical data; Tables S2 and S3: Chemical shifts of proline and cysteine residues in aqueous solution; Table S4: Structural statistics parameters calculated for synthetic PaD and Ct_PaD in aqueous solution; Table S5: Temperature coefficients for amide protons of PaD in aqueous solution.
