# Supplementary material for: The C-Terminus of Panusin, a Lobster β-Defensin, Is Crucial for Optimal Antimicrobial Activity and Serum Stability
Source: Pharmaceutics. 2023 Jun 20;15(6):1777. doi: 10.3390/pharmaceutics15061777 (PMC10301333; doi:10.3390/pharmaceutics15061777)
Supplement: Supplementary file 1 [file pharmaceutics-15-01777-s001.zip › pharmaceutics-2441242-supplementary.pdf]

pharmaceutics

MDPI

Supplementary Materials: The C-terminus of Panusin, a Lobster β-defensin, Is Crucial for Optimal Antimicrobial Activity and Serum Stability

Roberto Bello-Madruga, Javier Valle, M. Ángeles Jiménez, Marc Torrent, Vivian Montero-Alejoand David Andreu

1. Synthetic Peptide Characterization

Table S1. Peptide Analytical Data.

Peptide[1]	Theoretical Mass (Da)	Experimental Mass (Da)[2]	HPLC Retention Time (min)[3]	Purity (HPLC, %)[4]
PaD hexathiol	4265.76	4265.00	7.36	98.57
PaD	4259.76	4260.50	5.19	98.70
Ct_PaD tetrathiol	2805.26	2805.60	7.09	99.26
Ct_PaD	2801.25	2801.60	6.65	99.82

[1] All peptides are C-terminal carboxamides; [2] Determined by LC-MS, [3] Elution was carried out with a 10 to 50 linear gradient over 15 min; [4] Determined by analytical HPLC.

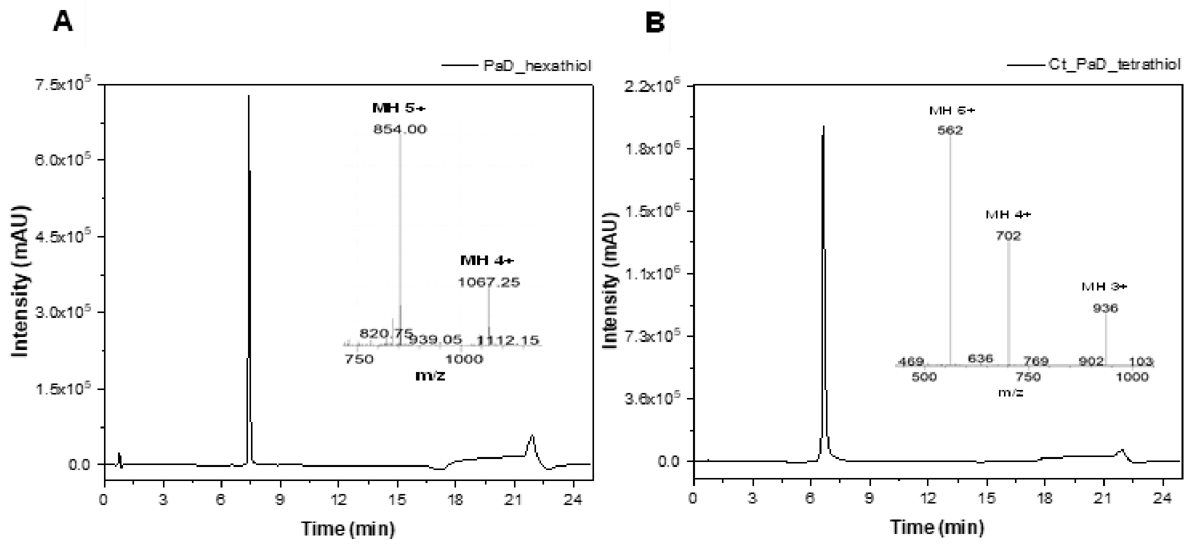

Figure S1. RP-HPLC elution profile of purified peptides. (Left) hexathiol precursor of synthetic PaD; inset, LC-MS spectrum: $[M + 5H]^{5+}$ (m/z 854) and $[M + 4H]^{4+}$ (m/z 1067.25) peaks are shown, and (Right) tetrathiol precursor of synthetic Ct_PaD; inset, LC-MS spectrum: $[M + 5H]^{5+}$ (m/z 562) and $[M + 4H]^{4+}$ (m/z 702) peaks are shown.

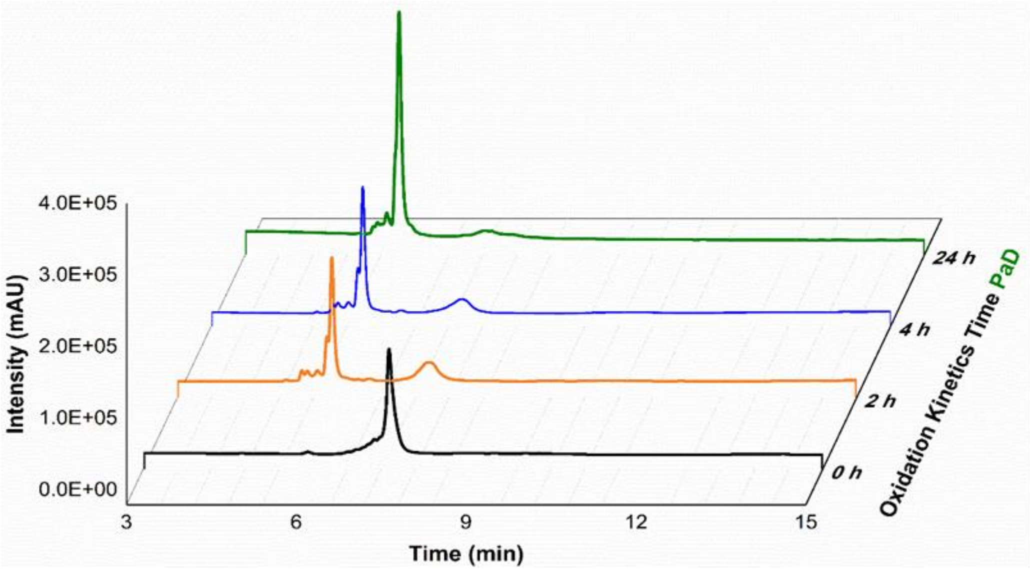

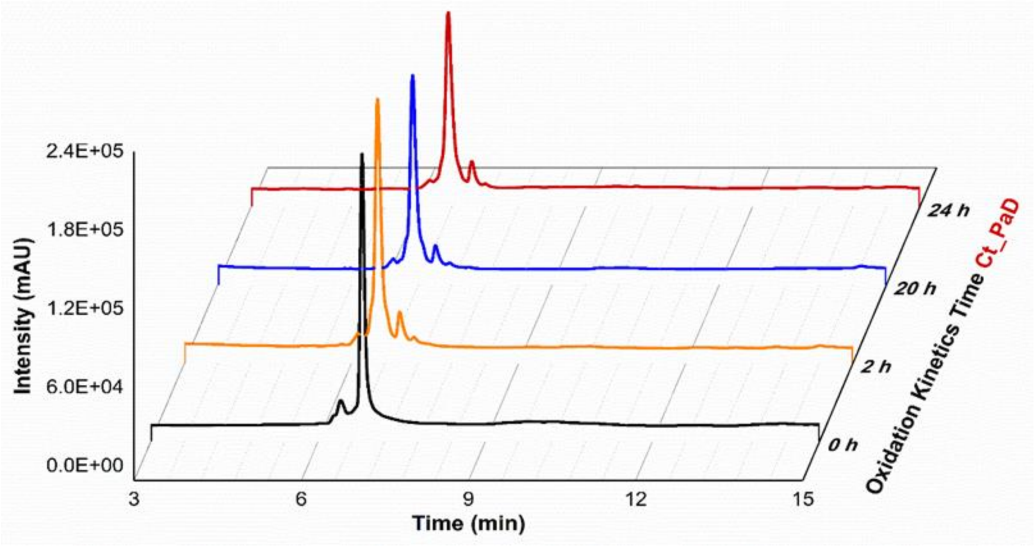

Figure S2. Oxidative Folding of peptides. RP-HPLC elution profile of kinetics oxidation time of PaD (Top) and Ct_PaD (Bottom).

2. Serum Stability Studies

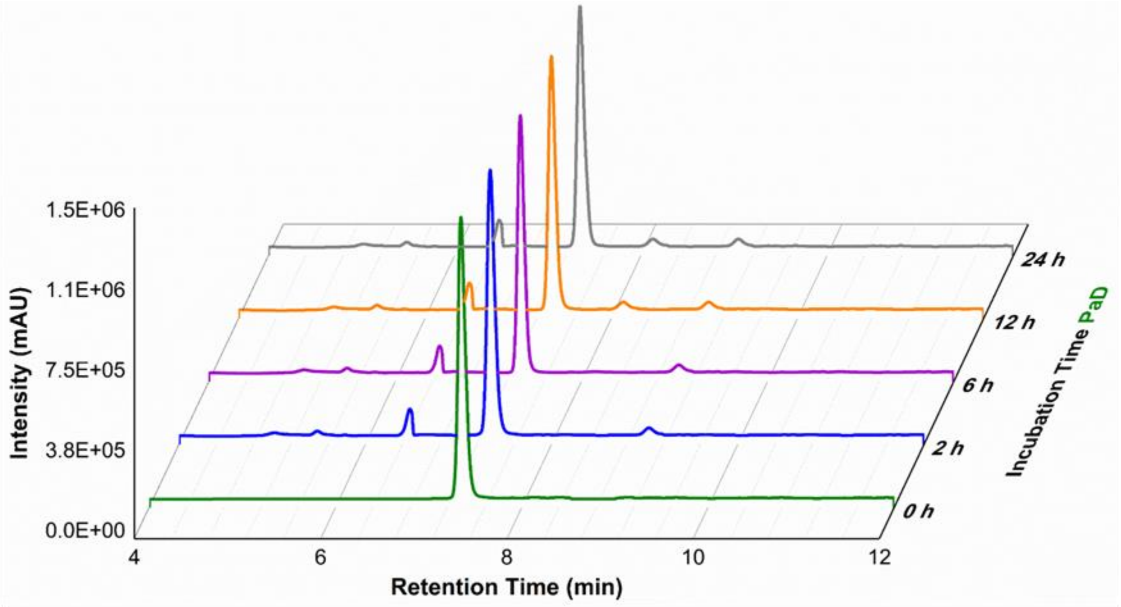

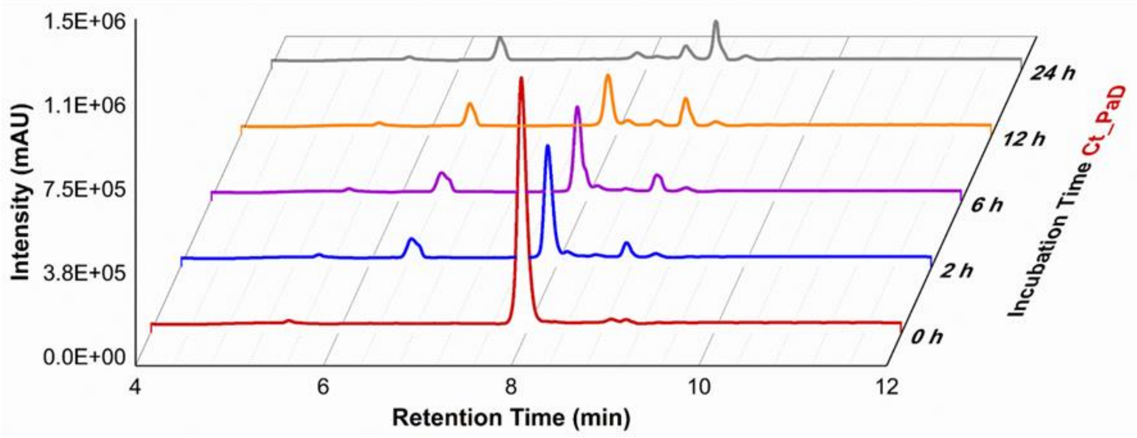

Figure S3. RP-HPLC chromatograms of peptides in human serum. Elution profiles after incubation of PaD (Top) and Ct_PaD (Bottom) with 50% (v/v) human serum at representative times (0, 2, 6, 12 and 24 h), using a 0% to 95% ACN gradient over 15 min. The major peaks correspond to the intact peptide.

3. NMR studies

Table S2. Differences in chemical shifts between the $^{13}C_{\beta}$ and $^{13}C_{\gamma}$ ($\Delta^{\beta\gamma} = \delta^{C\beta} - \delta^{C\gamma}$, ppm) for the Pro residues of PaD and Ct_PaD in aqueous solution at pH 5.5 and 25 °C.

PaD				Ct_PaD			
Residue	$\delta^{C\beta}$, ppm	$\delta^{C\gamma}$, ppm	$\Delta^{\beta\gamma}$, ppm	Residue	$\delta^{C\beta}$, ppm	$\delta^{C\gamma}$, ppm	$\Delta^{\beta\gamma}$, ppm
Pro 19	32.3	27.6	4.7	Pro 3	31.8	26.9	4.9
Pro 29	32.9	27.4	5.5	Pro 13	32.3	27.4	4.9

Table S3. Chemical shifts for the $^{13}C_{\beta}$ of the Cys residues of PaD and Ct_PaD in aqueous solution at pH 5.5 and 25 °C.

**PaD**		**Ct_PaD**	
Residue	$\delta^{C\beta}$, ppm	Residue	$\delta^{C\beta}$, ppm
Cys 6	39.9	--	--
Cys 13	39.0	--	--
Cys 18	42.4	Cys 2	40.1
Cys 28	43.8	Cys 12	41.3
Cys 34	36.9	Cys 18	41.8
Cys 35	45.9	Cys 19	40.0

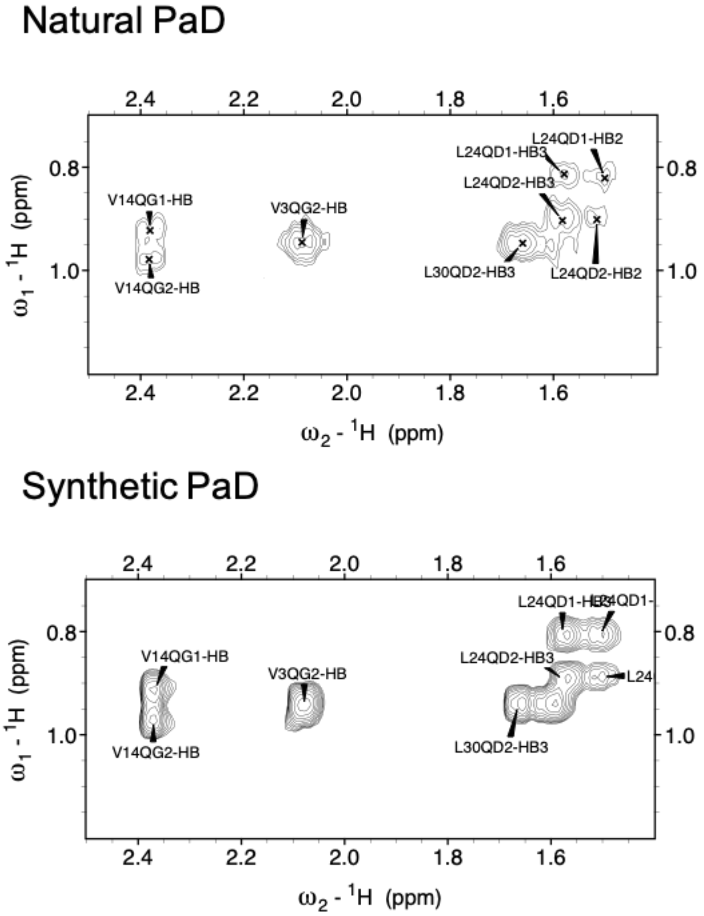

Figure S4. TOCSY spectra of natural and synthetic PaD. 2D ^{1}H,^{1}H-TOCSY of natural (top) and synthetic (bottom) PaD showing cross-peaks corresponding to Val and Leu methyl groups. Spectra were acquired in D_2O at 25 ºC.

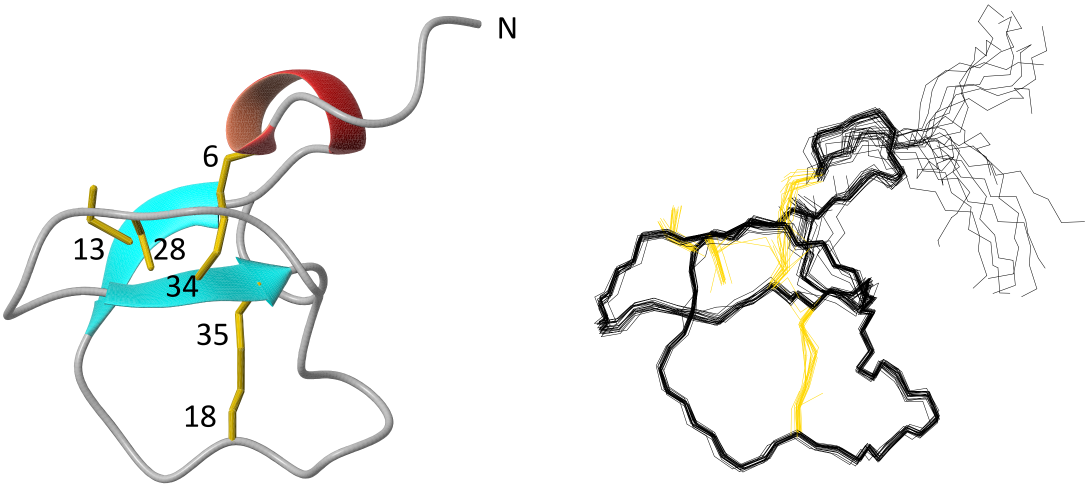

Figure S5. PaD structure calculated from NMR experimental without restrains for disulfide bond pairings**.** PaD structure calculated from NMR experimental restraints without including any restrains for disulfide bond pairings. (Left) Ribbon representation (Right) Overlay of backbone atoms (in black) of the 20 lowest target function conformers of the ensemble. Cys sidechains are in yellow.

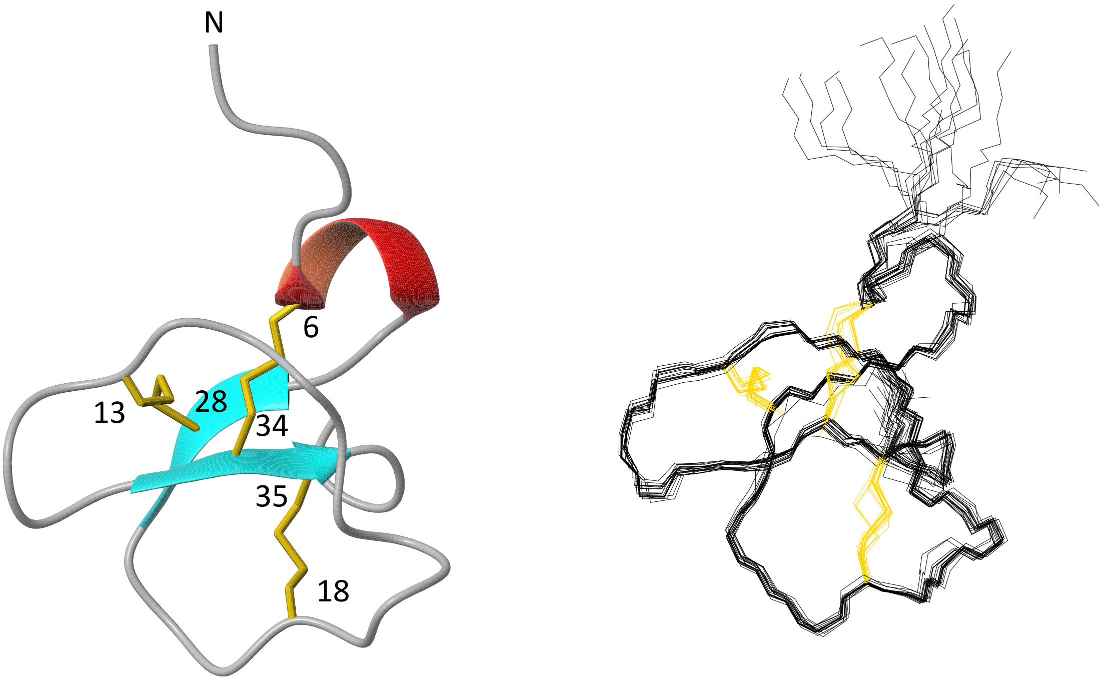

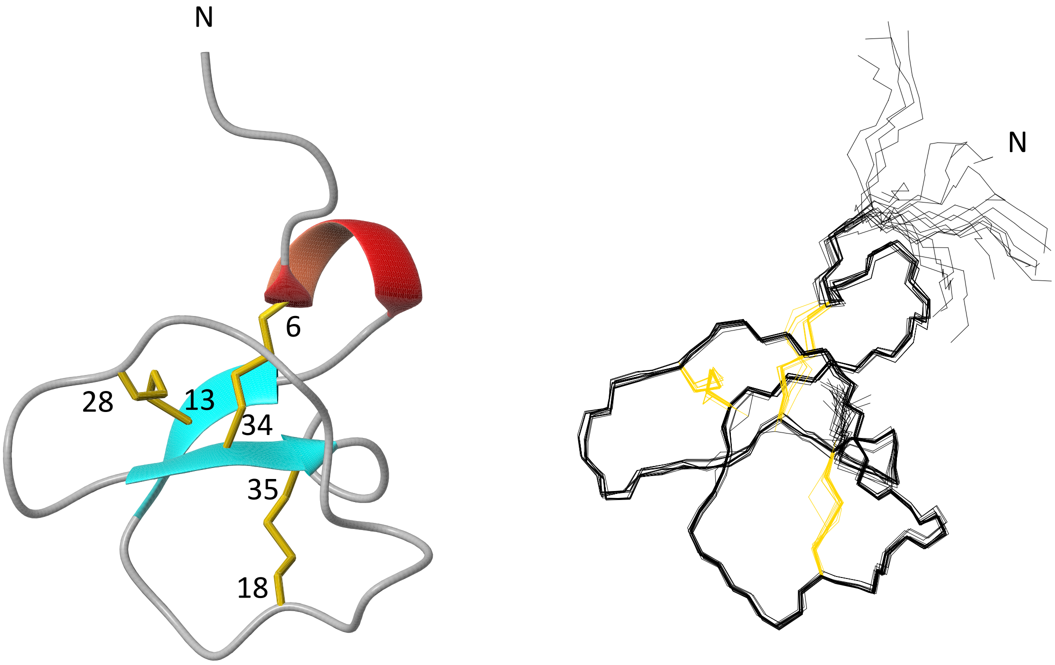

Figure S6. PaD structures calculated from NMR experimental restraints. PaD structures calculated from NMR experimental restraints. (Top) Including restrains for the disulfide bond pairings: 6-34, 13-28 and 18-35. (Bottom) Including restrains for the disulfide bond pairings: 6-34, 13-28 and 18-35, and for hydrogen-bonds. (Left panels) Ribbon representations. (Right panels) Overlay of backbone atoms (in black) of the 20 lowest target function conformers of the ensemble. Cys sidechains are in yellow.

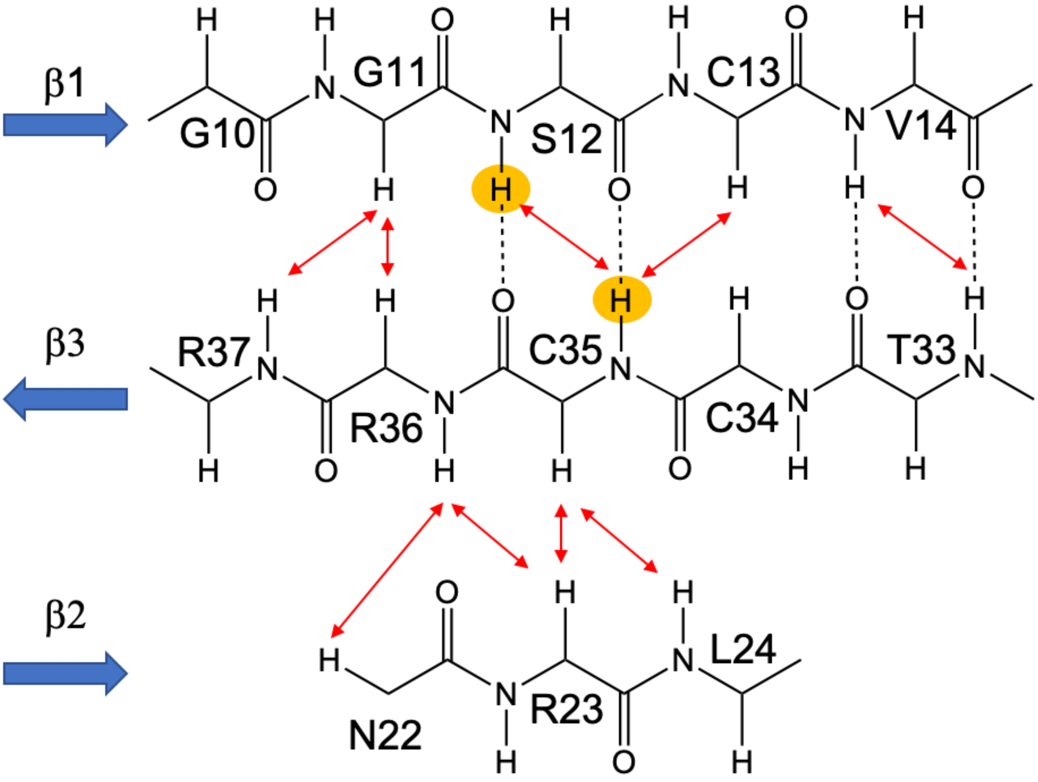

Figure S7. Schematic representation of the antiparallel β-sheet presents in the NMR structure of synthetic PaD. Schematic representation of the antiparallel β-sheet presents in the NMR structure of synthetic PaD. The red arrows connect protons for which NOE cross-peaks are present in NOESY spectra of PaD. Slow-exchanging HN amide protons are shown in a gold background.

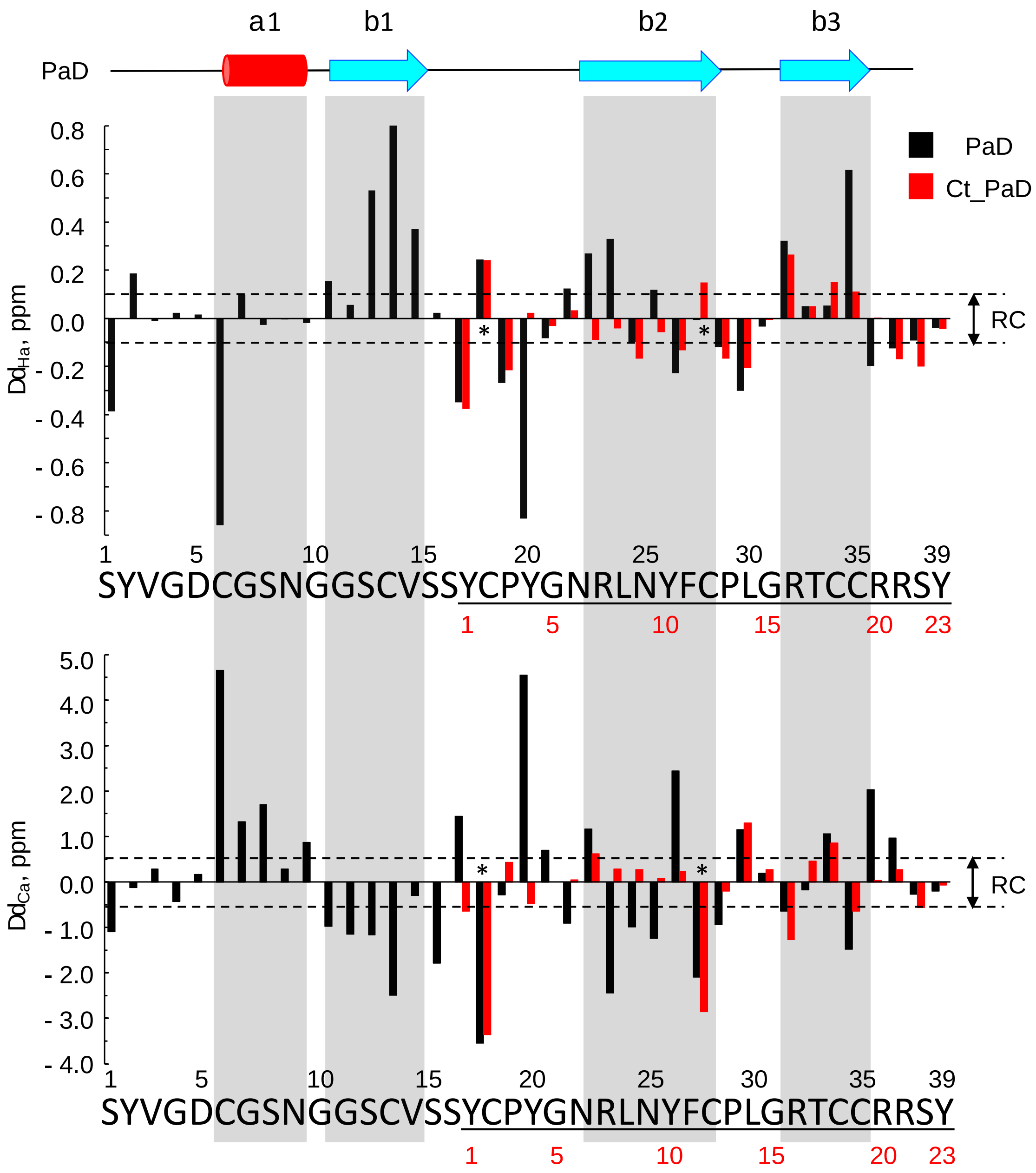

Figure S8. Conformational shifts of PaD and Ct_PaD as a function of sequence. Conformational shifts for the Hα protons ($\Delta\delta_{H\alpha} = \delta_{H\alpha}^{observed} - \delta_{H\alpha}^{RC}$, ppm) and Cα carbons ($\Delta\delta_{C\alpha} = \delta_{C\alpha}^{observed} - \delta_{C\alpha}^{RC}$, ppm) for PaD (black bars) and Ct_PaD (red bars) in aqueous solution at pH 5.5 and 25ºC as a function of sequence. $\delta_{H\alpha}^{RC}$ and $\delta_{C\alpha}^{RC}$ were taken from Wishart et al. 1995, *J. Biomol. NMR* 5, 67–81. Asterisks indicate Pro-preceding residues. The two dashed lines indicate the random coil range (RC). Secondary structure elements of PaD are schematically indicated at the top; helices are shown as red cylinders and sheets as cyan arrows. Note that negative $\Delta\delta_{H\alpha}$ and positive $\Delta\delta_{C\alpha}$ values are characteristics of helices, whereas β-sheets have positive $\Delta\delta_{H\alpha}$ and negative $\Delta\delta_{C\alpha}$ values.

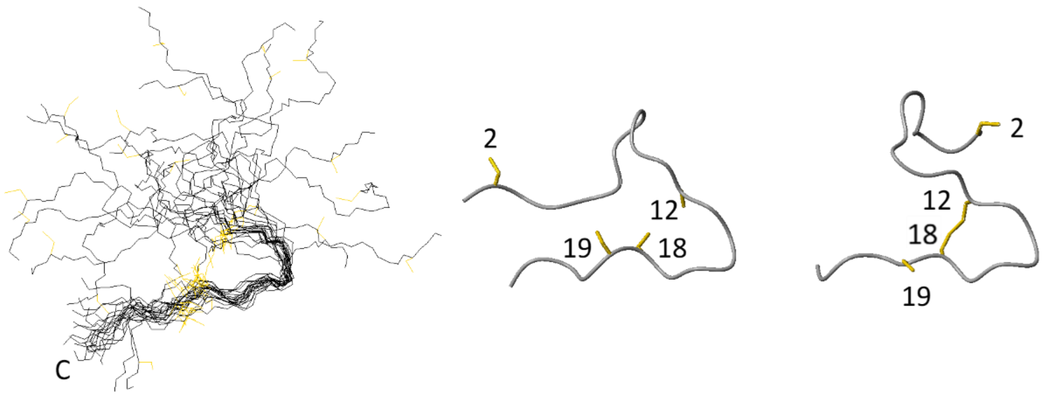

Figure S9. Ct_PaD structures calculated from NMR experimental without restrains for disulfide bond pairings**.** Ct_PaD structures calculated from NMR experimental restraints without including any restrains for disulfide bond pairings. (Left) Ribbon representation (Right) Overlay of backbone atoms (in black) of the 20 lowest target function conformers of the ensemble. Cys sidechains are in yellow.

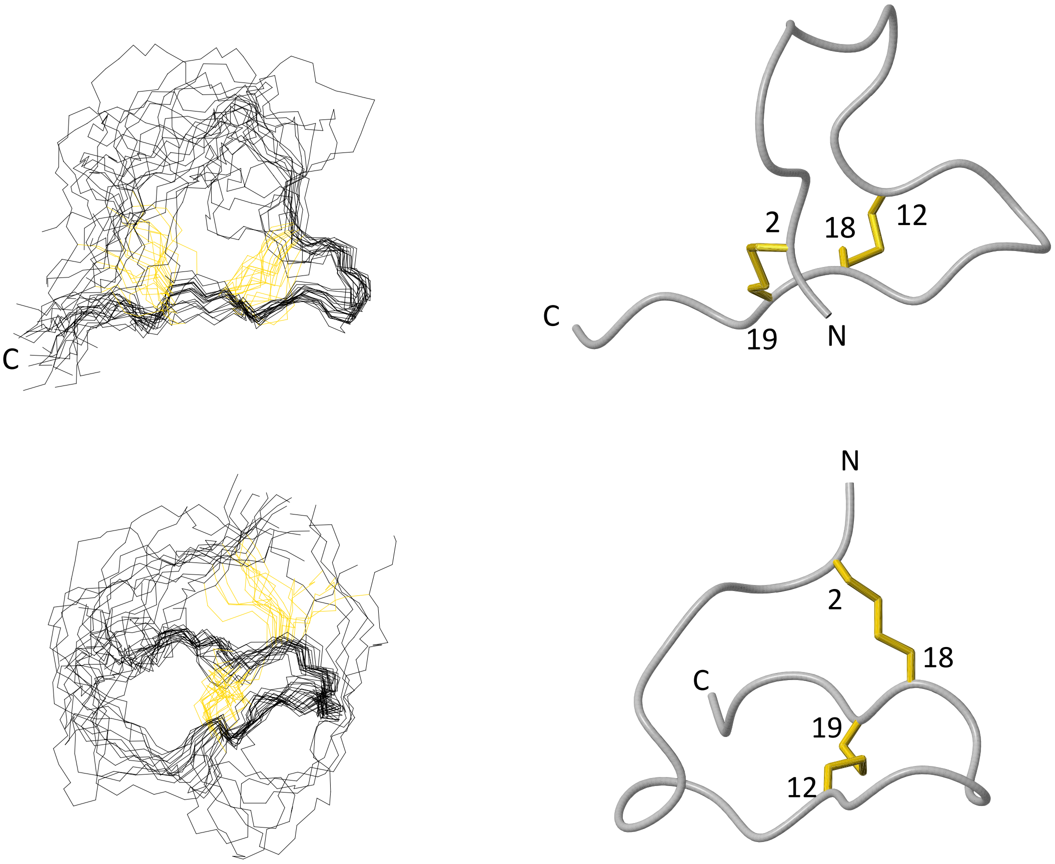

Figure S10. Ct_PaD structures calculated from NMR experimental restraints. Ct_PaD structures calculated from NMR experimental restraints. (Top) Including restrains for the disulfide bond pairings: 2-19, and 12-18. (Bottom) Including restrains for the disulfide bond pairings: 2-19, and 12-18. (Left panels) Ribbon representations. (Right panels) Overlay of backbone atoms (in black) of the 20 lowest target function conformers of the ensemble. Cys sidechains are in yellow.

Table S4. Structural statistics parameters calculated for synthetic PaD and Ct_PaD in aqueous solution. Summary of structural statistics parameters for the ensemble of the 20 lowest target function conformers calculated for synthetic PaD and Ct_PaD in aqueous solution.

Peptide	PaD[a]	PaD[b]	PaD[c]	Ct_PaD[a]	Ct_PaD[d]	Ct_PaD[e]
**Number of distance restraints**						
Intraresidue & sequential (i – j ≤ 1)	259	263	262	53	54	56
Medium range (1 < |i – j| < 5)	48	45	46	0	0	0
Long range (|i – j| ≥ 5)	57	59	57	0	0	0
Total number	364	367	365	53	54	56
Averaged total number per residue	9.3	9.4	9.4	2.3	2.3	2.4
**Number of dihedral angle constraints**						
Number of restricted ϕ angles	33	33	33	18	18	18
Number of restricted ψ angles	32	32	32	14	14	14
Total number	65	65	65	32	32	32
**Number of hydrogen bond restraints**	0	0	8	0	0	0
**Number of disulfide bond restraints**	0	3	3	0	2	2
Disulfide bond pairing	---	6–34<br>13–28<br>18–35	6–34<br>13–28<br>18–35	---	2–19<br>12–18	2–18<br>12–19
**Pairwise RMSD (Å)**						
**All residues**	**1-39**	**1-39**	**1-39**	**1-23**	**1-23**	**1-23**
Backbone atoms	1.8 ± 0.7	1.7 ± 0.8	1.7 ± 0.9	7.2 ± 1.9	3.5 ± 0.9	4.9 ± 2.8
All heavy atoms	2.4 ± 0.6	2.4 ± 0.7	2.5 ± 0.8	8.9 ± 1.9	5.4 ± 1.0	6.5 ± 2.7
**Ordered residues**	**5-38**	**5-38**	**5-38**	**12-22**	**12-22**	**12-22**
Backbone atoms	0.4 ± 0.1	0.5 ± 0.2	0.3 ± 0.1	1.8 ± 0.6	1.3 ± 0.5	1.3 ± 0.6
All heavy atoms	1.0 ± 0.2	1.1 ± 0.2	1.1 ± 0.2	3.2 ± 0.8	2.7 ± 0.7	2.8 ± 0.8
**Ramachandran plot (%)**						
Most favoured regions	85.5	78.4	83.1	77.1	75.0	70.0
Additionally allowed regions	14.5	21.6	16.9	22.9	24.4	29.7
Generously allowed regions	0	0	0	0	0	0.3
Disallowed regions	0	0	0	0	0	0

[a] Data for structure calculation without restraints for disulfide bond pairings. [b] Data for structure calculation including restraints for disulfide bond pairings, but not for hydrogen-bond. [c] Data for structure calculation including restraints for disulfide bond pairings and for hydrogen-bonds. [d] Data for structure calculation including restraints for disulfide bond pairings, i.e. 2-19 & 12-18. [e] Data for structure calculation including restraints for disulfide bond pairings, i.e. 2-18 & 12-19.

Table S5. Temperature coefficients for amide protons of PaD in aqueous solution.

Residue	$\Delta\delta/\Delta T$, 10^3 ppm.K^{-1}	Residue	$\Delta\delta/\Delta T$, 10^3 ppm.K^{-1}	Residue	$\Delta\delta/\Delta T$, 10^3 ppm.K^{-1}
Ser 1	---	Val 14	-3.0	Phe 27	-4.8
Tyr 2	-5.8	Ser 15	-7.9	Cys 28	-4.2
Val 3	-7.7	Ser 16	-1.7	Pro 29	---
Gly 4	-3.0	Tyr 17	-9.2	Leu 30	-4.3
Asp 5	-5.3	Cys 18	-10.2	Gly 31	-6.8
Cys 6	-7.2	Pro 19	---	Arg 32	-5.8
Gly 7	-3.1	Tyr 20	-7.5	Thr 33	-4.1
Ser 8	-3.8	Gly 21	-7.8	Cys 34	-3.5
Asn 9	-1.5	Asn 22	-2.6	Cys 35	-1.3
Gly 10	-4.1	Arg 23	-1.1	Arg 36	-5.6
Gly 11	-3.8	Leu 24	-2.6	Arg 37	-5.7
Ser 12	-1.3	Asn 25	-7.1	Ser 38	-7.2
Cys 13	-6.2	Tyr 26	-6.0	Tyr 39	-6.2
